# Correction to: Two-year outcome after recurrent implantation failure: prognostic factors and additional interventions

**DOI:** 10.1007/s00404-018-4873-7

**Published:** 2018-08-22

**Authors:** Christiane Kling, Andreas Schmutzler, Georg Wilke, Jürgen Hedderich, Dieter Kabelitz

**Affiliations:** 1 Institute of Immunology, University Medical Center Schleswig-Holstein, Michaelisstr. 5, 24105 Kiel, Germany; 2 Department of Obstetrics and Gynaecology, IVF Unit, University Medical Center Schleswig-Holstein, Kiel, Germany; 3 Fertility Center, Hildesheim, Germany; 4 Department of Medical Informatics and Statistics, University Medical Center Schleswig-Holstein, Kiel, Germany

## Correction to: Arch Gynecol Obstet (2008) 278:135–142 http://doi.org/10.1007/s00404-007-0538-7

The article Two-year outcome after recurrent implantation failure: prognostic factors and additional interventions, written by Christiane Kling, Andreas Schmutzler, Georg Wilke, Jürgen Hedderich, Dieter Kabelitz, was originally published electronically on the publisher’s internet portal (currently SpringerLink) on 12 January 2008 without open access. With the author(s)’ decision to opt for Open Choice the copyright of the article changed on 24 August 2018 to © The Author(s) 2018 and the article is forthwith distributed under the terms of the Creative Commons Attribution 4.0 International License (http://creativecommons.org/licenses/by/4.0/), which permits use, duplication, adaptation, distribution and reproduction in any medium or format, as long as you give appropriate credit to the original author(s) and the source, provide a link to the Creative Commons license and indicate if changes were made.

